# Obesity and Vitamin D Insufficiency among Adolescent Girls and Young Adult Women from Korea

**DOI:** 10.3390/nu11123049

**Published:** 2019-12-13

**Authors:** Haeun Jang, Yujin Lee, Kyong Park

**Affiliations:** Department of Food and Nutrition, Yeungnam University, 280 Daehak-ro, Gyeognsan, Gyeongbuk 38541, Korea; janghaeun@ynu.ac.kr (H.J.); yj_lee@yu.ac.kr (Y.L.)

**Keywords:** Vitamin D insufficiency, adolescents, women, obesity, Korea

## Abstract

Although there is evidence of the biological mechanisms by which obesity may induce vitamin D insufficiency or deficiency, limited epidemiological studies have been conducted, especially among Asian adolescent girls and young adult women who are at a high risk of vitamin D deficiency. This study aimed to examine the cross-sectional association between obesity and vitamin D insufficiency among adolescent girls and young adult women in Korea. We used data from the Korea National Health and Nutrition Examination Survey (KNHANES) 2008–2014, and 3623 girls and young adult women aged 12–29 years were included. Demographic and lifestyle data were collected using a self-administered questionnaire from the health interview survey. Serum 25-hydroxyvitamin D (25(OH)D) level, body mass index (BMI), and body fat percentage (BF%) were measured during health examinations. Multivariable logistic regression was used considering the complex, multistage probability sample design of KNHANES. In the multivariable-adjusted analyses, obese girls and women, defined by BMI, were more likely to have a higher prevalence of vitamin D insufficiency (odds ratio [OR]: 1.49, 95% confidence interval [CI]: 1.03–2.17). This association was also evident for BF%. Participants with ≥30% BF% had a significantly higher prevalence of vitamin D insufficiency (OR: 1.52, 95% CI: 1.07–2.16). Obesity may worsen vitamin D insufficiency among adolescents and young women because of the fat-soluble characteristics of vitamin D and related health behaviors, such as a lack of outdoor activity. Further large-scale prospective cohort studies or randomized controlled trials are warranted to confirm this causality.

## 1. Introduction

Vitamin D can be supplied via food or supplements and can also be synthesized in the skin by the action of ultraviolet B light; however, the prevalence of vitamin D insufficiency or deficiency has been increasing and has become an emerging public health issue, particularly among young Asian women [1,2,3]. According to a meta-analysis of studies on vitamin D deficiencies, there are racial or regional differences in vitamin D status. For example, the level of 25-hydroxyvitamin D(25(OH)D), a proxy for vitamin D status, was generally higher in North America than in other countries, such as Europe, the Asia Pacific region, the Middle East, Africa, or South America [4]. In the meta-analysis, an age-related difference was observed in the Asian population, where children and adolescents had lower serum 25(OH)D levels than did individuals in other age groups; however, there was no difference between age groups in North America and Europe. In contrast, children and adolescents in the Middle East and Africa had higher 25(OH)D levels than did adults [4].

Korean women are more likely to have vitamin D insufficiency or deficiency than Korean men [5,6,7]. Similar to the trend observed in other Asian countries, the prevalence of vitamin D insufficiency or deficiency was much higher in adolescent girls and young adult women than other age groups, even the elderly [5,7]. Epidemiologic studies have shown that vitamin D insufficiency or deficiency is closely associated with chronic diseases, such as tumors, cardiovascular diseases, and diabetes [8,9,10,11,12,13]. Indeed, vitamin D is fat soluble, meaning that it can be stored in body fat (BF) tissues, and excessive BF can reduce 25(OH)D levels in the body, especially in obese individuals. A recent meta-analysis of observational studies revealed that obese participants were more likely to have vitamin D deficiency, regardless of age or latitude [14]. However, limited studies have assessed the association between obesity and 25(OH)D levels in Asian adolescent girls and young adult women who are at a high risk for vitamin D insufficiency or deficiency.

Therefore, this study aimed to investigate the association between obesity and vitamin D status among Korean adolescent girls and young adult women by analyzing data from the Korea National Health and Nutrition Examination Survey (KNHANES).

## 2. Materials and Methods

### 2.1. Study Population

KNHANES is a nationwide, representative, and cross-sectional survey that has assessed the health and nutritional status of the non-institutionalized civilian population of Korea since 1998. Its versions include KNHANES I (1998), II (2001), III (2005), IV (2007–2009), V (2010–2012), VI (2013–2015), VII (2016–2018), and VIII (2019–ongoing). KNHANES comprises three parts: a health interview, a nutritional examination, and a health examination. More detailed information on KNHANES is provided elsewhere [15].

KNHANES data are collected and managed by the Korea Centers for Disease Control and Prevention (KCDC), and this study was approved by the Ethics Committee of the KCDC (Approval numbers: 2008-04EXP-01-C, 2009-01CON-03-2C, 2010-02CON-21-C, 2011-02CON-06-C, 2012-01EXP-01-2C, 2013-07CON-4C, and 2013-12EXP-03-5C). Before data collection, all participants submitted written informed consent.

The present study used KNHANES data from 2008 to 2014, where data on serum 25(OH)D are available. Of the 5899 women aged 12–29 years who were eligible for inclusion in the present study, women who had missing data on their serum 25(OH)D levels (*n =* 1451) or body mass index (BMI) values (*n =* 15), missing values on sampling weight (*n =* 607), an implausible total energy intake (<500 kcal or >5000 kcal) (*n =* 53) [16], or were pregnant or breastfeeding (*n =* 150) at the time of the survey were excluded. Ultimately, 3623 women were included.

### 2.2. General Characteristics

For the analysis, household income levels were divided into low to mid-low and mid-high to high. Education level was reclassified as middle school graduation or lower and high school graduation or higher. The metabolic equivalents were calculated by multiplying the frequency and duration of activities by assigning weights according to intensity levels (walking, moderate, and high intensity) and were divided into quartiles. Smoking status was categorized into non-smokers and current smokers, while alcohol consumption was classified into non-alcohol drinkers and alcohol drinkers depending on the frequency of drinking during the past year. The frequency of eating-out was reclassified as <4 times/week, 5–6 times/week, 1 time/day, and ≥2 times/day.

### 2.3. Measurement of Serum 25(OH)D Levels

Blood samples were collected after at least 12 h of fasting. As a surrogate marker for vitamin D status, serum 25(OH)D was measured using the 1470 Wizard gamma counter (Perkin Elmer, Turku, Finland) with a 25-hydroxyvitamin D radioimmunoassay kit (DiaSorin Inc, Stillwater, OK, USA) at the central testing institute center (NeoDin Medical Institute, Seoul, Korea). Serum 25(OH)D levels were categorized into insufficiency and sufficiency groups based on a 20 ng/mL limit, according to the criteria provided by the Institute of Medicine [17]. Detailed information on the validity of this instrument is available elsewhere [5].

### 2.4. Measurement of Obesity

Obesity status was measured based on BMI and BF percentage (BF%). Height and weight were measured by a trained nurse and staff. BMI was calculated as body weight in kg divided by height in meters squared. BF was assessed in the subsample of participants, including women aged ≥19 years, from September 2008 to June 2009, and in women aged ≥10 years from July 2009 to May 2011, using dual-energy X-ray absorptiometry (DXA; QDR 4500A, Hologic Inc., Bedford, MA, USA).

Obesity was defined differently in each age group. For young adult women, we used the World Health Organization criteria for the Asian population, which defines obesity as a BMI ≥25 kg/m^2^ [18]. Obesity among female adolescents was defined as being above the 95th percentile of the age-specific BMI percentile based on the 2017 Pediatric Growth Chart [19]. We also defined obesity as BF% ≥30%, based on the criteria of the Korean Society of Obstetrics and Gynecology [20].

### 2.5. Statistical Analysis

To integrate KNHANES data from 2008 to 2014, a complex, multistage probability sample design analysis was performed considering the primary sampling unit, stratification variables, and weight. The demographic characteristics between groups were compared using a chi-squared test or t-test. A multivariable logistic regression analysis was used to calculate the odds ratio (OR) and 95% confidence intervals (CIs). Based on a preliminary analysis and literature review, potential confounding factors and effect modifiers were considered in the study analysis [16,21,22,23,24,25]. We found no significant effect modifier for the association between obesity and vitamin D status. A statistical analysis was performed using SAS (Statistical Analysis System version 9.4, SAS Institute, Cary, NC, USA), and statistical significance was set at α = 0.05.

## 3. Results

The general characteristics of the participants according to obesity status (defined by BMI) are shown in Table 1. Approximately 12% of participants were obese; the average serum vitamin 25(OH)D levels were 15.11 ± 5.17 ng/mL in non-obese women and 14.70 ± 4.79 ng/mL in obese women. The current smoking rates were very low (6–11%), but more than half of the women reported that they had consumed alcohol during the past year. Approximately 36% and 29% of the non-obese and obese women, respectively, reported eating outside the home at least once a day.

Table 2 presents the vitamin D and obesity status among adolescent girls and young adult women. Compared with adolescents, young adult women were more likely to a have higher rate of obesity, defined by the BMI percentiles for adolescent girls and the BMI cut-offs for young adult women, as well as lower serum vitamin 25(OH)D levels. There were no differences in the BF% in these unadjusted comparisons between adolescent girls and young adult women.

The association between obesity and vitamin D insufficiency is shown in Table 3. After adjustment for multiple potential confounding factors, BMI-diagnosed obesity was inversely associated with vitamin D insufficiency (OR: 1.49, 95% CI: 1.03–2.17). Similarly, BF%-diagnosed obesity was inversely associated with vitamin D insufficiency (OR: 1.52, 95% CI: 1.07–2.16).

The association between obesity and vitamin D sufficiency was further analyzed by stratifying the age group (Table 4). There were no significant differences in the associations of BMI-diagnosed obesity and BF%-diagnosed obesity with vitamin D insufficiency when stratified by age (the *P* interaction was 0.90 for BMI-diagnosed obesity and 0.97 for BF%-diagnosed obesity).

## 4. Discussion

This study investigated the association between obesity and vitamin D insufficiency among adolescent girls and young adult women aged 12–29 years using integrated data from the 2008–2014 KNHANES. The prevalence of obesity, assessed based on BMI cut-off/BMI percentiles and BF%, was inversely associated with vitamin D insufficiency.

Our findings are consistent with those of other cross-sectional studies among adolescents and young adult women [26,27]. The 25(OH)D levels among US adolescents had a significant negative association with BMI percentile and BF% [27]. In addition, the BF mass in the vitamin D deficiency group was approximately 5 kg higher than that in the vitamin D sufficient group [26]. Furthermore, the results from the National Health and Nutrition Examination Survey in the United States indicated that among premenopausal white women, the odds of hypovitaminosis D in the obese group with BMI ≥30 kg/m^2^ are 3.3 times higher than the odds in the normal weight group with BMI 18.5–25 kg/m^2^ (OR: 3.3; 95% CI: 1.6–7.2) [28]. A review article of 23 studies of pediatric populations described that a very high prevalence of vitamin D insufficiency is observable among overweight and obese children [29]. Similarly, a recent meta-analysis indicated that vitamin D deficiency is more common among obese participants and that obesity-vitamin D deficiency association is stronger in the Asian population than in the European–American population (OR 3.70, 95% CI 1.98–6.90 vs. OR 3.09, 95% CI 1.89–5.04) [30].

Although many studies have identified an inverse association between low vitamin D status and obesity or between obesity and vitamin D insufficiency or deficiency, it remains unclear whether low vitamin D status is responsible for the development of obesity or whether obesity results in vitamin D insufficiency or deficiency. Besides low intake, a low cutaneous synthesis of vitamin D or fewer outdoor activities among obese participants are possible hypotheses that have been suggested as relevant mechanisms. For example, vitamin D sequestered in adipose tissue can lead to lower serum 25(OH)D levels, especially in obese individuals [31]. However, the explanation of the effect of 25(OH)D levels on BF mass has not been sufficiently explored [32]. Therefore, further research is needed to clarify the mechanism of action in humans.

This study has several strengths and limitations. First, the possibility of residual confounding cannot be excluded. For example, the use of a UV blocking cream, time of sun exposure, or season in which serum 25(OH)D levels were measured were not considered in the analysis due to a lack of data. These factors are important, as they may affect young adult women at a high risk of vitamin D insufficiency or deficiency. Second, we identified the association between obesity and vitamin D status. However, a cross-sectional study has inherent problems in identifying causal relationships between exposure and outcome. Therefore, further investigations, such as well-designed longitudinal studies and clinical trials, are needed to confirm this causality.

The notable strength of this study is the integration of the 7-year KNHANES data, which features a nationally representative sample of Koreans, thereby allowing the study’s findings to be generalized. Second, obesity was assessed based on the BMI/BMI percentile and BF% with high accuracy and reproducibility. Height and weight were measured by trained personnel, and BF% was assessed using DXA, which is the gold standard method. In addition, serum 25(OH)D levels were assessed at the same central institute for 7 years to minimize the heterogeneity of measurement. Given the methodological strengths and the quality of data used in this study, the present results can serve as a basis for the development of vitamin D insufficiency prevention and management policies targeted at the overweight and obese population. These findings respond to what is currently a significant public health burden among young Asian women.

## 5. Conclusions

The prevalence of vitamin D insufficiency or deficiency among adolescent girls and young adult women in Korea is considerably high, and the serum 25(OH)D levels of these women are inversely associated with obesity. Consequently, it is likely that obesity may worsen the vitamin D status in this age group. This finding could form a scientific basis for the management and prevention of vitamin D insufficiency in adolescents and young adult women, particularly women with obesity.

## Figures and Tables

**Table 1:** Participants’ characteristics stratified by obesity status.

	Non-Obese	Obese ^1)^
*N* (%)	3194 (88)	429 (12)
Age, years	19.93 ± 5.45	21.47 ± 5.48
Body fat percentage, %	30.67 ± 5.12	39.00 ± 4.06
Serum vitamin 25(OH) D, ng/mL	15.11 ± 5.17	14.70 ± 4.79
Current smokers	173 (6)	43 (11)
Alcohol drinkers	1796 (57)	270 (64)
Household income		
Low/Mid–low	1112 (35)	196 (46)
Mid–high/High	2050 (65)	230 (54)
Education level		
Middle school graduation or lower	1383 (44)	146 (34)
High school graduation or higher	1782 (56)	278 (66)
Physical activity (METs)		
Low	756 (26)	73 (19)
Mid–low	751 (26)	76 (20)
Mid–high	737 (25)	95 (25)
High	687 (23)	141 (37)
Frequency of eating-out		
<4 times/week	634 (20)	143 (33)
5–6 times/week	1418 (45)	160 (37)
1 time/day	725 (23)	89 (21)
≥2 times/day	408 (13)	36 (8)
Energy-yielding nutrient intake		
Total energy, kcal	1812.65 ± 697.22	1700.21 ± 667.03
Energy from carbohydrate, %	61.89 ± 11.42	61.49 ± 11.80
Energy from protein, %	14.36 ± 4.23	14.80 ± 4.52
Energy from fat, %	22.95 ± 8.72	22.94 ± 9.08

Values are *n* (%) or the mean ± standard deviation. ^1)^ Obesity was defined using BMI cut-offs ≥95th percentile for adolescent girls and ≥25 kg/m^2^ for young adult women. METs, Metabolic Equivalent of Tasks.

**Table 2:** Vitamin D and obesity status among adolescent girls and young adult women.

	Adolescent Girls (12–18 Years)	Young Adult Women (19–29 Years)	*p*
*N* (%)	1577 (44)	2046 (56)	
Age	14.69 ± 1.94	24.29 ± 3.19	<0.0001
Serum vitamin 25(OH) D, ng/mL			
Mean ± SD (Min, Max)	15.63 ± 5.12 (4.64, 46.30)	14.62 ± 5.10 (4.44, 42.44)	<0.0001
Vitamin D sufficiency	292 (19)	258 (13)	<0.0001
Vitamin D insufficiency	1285 (81)	1788 (87)	
Body mass index, kg/m^2^			
Mean ± SD (Min, Max)	20.64 ± 3.30 (12.65, 39.14)	21.45 ± 3.63 (14.38, 48.96)	<0.0001
<95th percentiles or <25 kg/m^2^	1435 (90)	1759 (86)	<0.0001
≥95th percentiles or ≥25 kg/m^2^	142 (9)	287 (14)	
Body fat percentage, %			
Mean ± SD (Min, Max)	31.98 ± 5.90 (14.68, 51.55)	31.58 ± 5.61 (17.71, 53.79)	0.1776
<30%	198 (38)	417 (40)	0.4775
≥30%	326 (62)	635 (60)	

**Table 3:** Association between obesity and vitamin D insufficiency ^1)^.

	Obesity ^2)^		≥30% of BF% ^3)^	
	Non-Obese	Obese	Non-Obese	Obese
*N*	3194	429	615	961
No. of cases	2697	376	495	830
Model 1	Reference	1.26 (0.88–1.81)	Reference	1.47 (1.04–2.06)
Model 2	Reference	1.46 (1.00–2.13)	Reference	1.50 (1.05–2.14)
Model 3	Reference	1.49 (1.03–2.17)	Reference	1.52 (1.07–2.16)

BMI, body mass index; BF%, body fat percentage. Values are expressed as the odds ratios (95% confidence intervals). ^1)^ Model 1: age-adjusted; Model 2: Model 1 plus additional adjustments for smoking status, alcohol consumption, education, household income, total energy intake, and physical activity; and Model 3: further adjusted for the frequency of eating-out. ^2)^ Obesity was defined using BMI cut-offs ≥95th percentile for adolescent girls and ≥25 kg/m^2^ for young adult women. ^3)^ Data on BF% are available from KNHANES 2008–2011, and the missing data were *n* = 2047.

**Table 4:** Association between obesity and vitamin D insufficiency by age group ^1)^.

12–18 Years	Obesity ^2)^		≥30% of BF% ^3)^	
	Non-Obese	Obese	Non-Obese	Obese
*N*	1435	142	198	326
No. of cases	1163	122	157	279
Model 1	Reference	1.26 (0.88–1.81)	Reference	1.47 (1.04–2.06)
Model 2	Reference	1.45 (0.82–2.57)	Reference	1.55 (0.91–2.65)
Model 3	Reference	1.56 (0.83–2.96)	Reference	1.57 (0.92–2.68)
**19–29 Years**	**Obesity ^2)^**		**≥30% of BF%** **^3)^**	
	**Non-Obese**	**Obese**	**Non-Obese**	**Obese**
*N*	1759	287	417	635
No. of cases	1534	254	338	551
Model 1	Reference	1.16 (0.73–1.84)	Reference	1.47 (1.04–2.06)
Model 2	Reference	1.43 (0.90–2.28)	Reference	1.49 (1.02–2.17)
Model 3	Reference	1.48 (0.94–2.34)	Reference	1.55 (1.05–2.29)
*p* for interaction	0.90		0.97	

BMI, body mass index; BF%, body fat percentage. Values are expressed as the odds ratios (95% confidence intervals). ^1)^ Model 1: age-adjusted; Model 2: Model 1 plus additional adjustments for smoking status, alcohol consumption, education, household income, total energy intake, and physical activity; and Model 3: further adjusted for the frequency of eating-out. ^2)^ Obesity was defined using BMI cut-offs ≥95th percentile for adolescent girls, and ≥25 kg/m^2^ for young adult women. ^3)^ Data on BF% are available from KNHANES 2008–2011, and the missing data were *n* = 2047.
